# The Erector Spinae Plane Block with 20 or 30 mL of 0.25% Bupivicaine Provides Equivalent Postoperative Analgesia after Mastectomy: A Prospective Randomized Trial

**DOI:** 10.4274/TJAR.2024.241730

**Published:** 2025-02-11

**Authors:** Merve Bıdak, Bahadır Çiftçi, Pelin Basım, Birzat Emre Gölboyu, Yunus Oktay Atalay

**Affiliations:** 1 İstanbul Medipol University Faculty of Medicine, Department of Anaesthesiology and Reanimation, İstanbul, Türkiye; 2 İstanbul Medipol University Faculty of Medicine, Department of General Surgery, İstanbul, Türkiye; 3 İzmir Katip Çelebi University Faculty of Medicine, Department of Anaesthesiology, İzmir, Türkiye

**Keywords:** Breast, erector spinae plane block, mastectomy, pain management, volume

## Abstract

**Objective:**

Analgesia management following breast surgery is a critical concern. The erector spinae plane block (ESPB) is a regional anaesthesia technique that is frequently used for analgesia after breast surgery. However, there is no consensus on the volume. Therefore, the aim of this study was to compare ESPB performed using 20 mL vs. 30 mL.

**Methods:**

The study included 43 female patients with American Society of Anesthesiologist class I-II physical status. Participants were randomized into two groups: 20 mL ESPB and 30 mL ESPB. Ibuprofen (400 mg) 3x1 was ordered, and a fentanyl patient-controlled analgesia device was attached intravenously to the participants. If the pain score was ≥4, meperidine (0.5 mg kg^-1^) was administered.

**Results:**

Postoperative fentanyl use was similar between the groups. There was no difference in the amount of rescue analgesic use between the groups. The static and dynamic numerical rating scores were similar between the groups. No statistical difference was noted in terms of nausea, vomiting, or itching between the groups.

**Conclusion:**

A similar analgesic effect is achieved by performing ESPB using 20 or 30 mL of local anaesthetic at the same concentration.

**Main Points**

• Erector spinae plane block (ESPB) is a regional anaesthesia method frequently used for analgesia management after breast surgery.

• However, different spread patterns have been reported in radiological and cadaver studies of this block.

• The amount of volume to be applied is also a matter of debate.

• In this study, we observed that 20 and 30 mL ESPB had equal analgesic efficacy.

## Introduction

Pain control is a significant challenge that affects patient comfort after mastectomy and axillary lymph node dissection, which are common surgeries nowadays. In the postoperative period, approximately 50% of patients experience severe acute pain that may lead to the development of chronic pain.^1^ This pain can hinder postoperative recovery by causing difficulties in breathing and delayed mobilization,^1, 2^ and regional anaesthesia methods, such as the erector spinae plane block (ESPB), are employed to manage postoperative pain following breast surgery.

When applied at the T4-T5 level, ESPB provides thoracic analgesia by targeting the posterior and anterior branches of the spinal nerves through the administration of a local anaesthetic between the transverse process (TP) of the thoracic vertebra and the anterior fascia of the erector spinae muscles (ESM).^3^ Because the block’s application site is distant from the pleura and neuraxial tissues, it has a reduced risk of complications related to injury to these structures. Performing the block under ultrasound guidance allows visualization of the anatomy and spread of the local anaesthetic.^3, 4, 5^

Randomized controlled studies have reported that ESPB provides effective postoperative analgesia after breast surgery.^6, 7, 8, 9, 10, 11, 12, 13, 14, 15, 16, 17, 18^ However, the optimal volume of local anaesthetic to be administered remains unclear. Radiological imaging and cadaver studies have demonstrated different spread patterns with varying local anaesthetic volumes.^11, 12, 13, 14^ Therefore, in our study, we performed ESPB with different volumes of local anaesthetic (20 mL and 30 mL) to evaluate the effectiveness of pain relief in participants who underwent mastectomy and axillary lymph node dissection. Our primary goal was to compare opioid consumption after surgery, with secondary goals of assessing and comparing postoperative pain levels [numerical rating score (NRS)], the amount of rescue analgesic use, and the adverse consequences of opioid use, including emesis, nausea, and allergic responses.

## Methods

### Study Design

This prospective, randomized study was approved by the İstanbul Medipol University Non-Interventional Clinical Research Ethics Committee (approval no.: 997, dated: September 30, 2021). After receiving ethics committee approval, the study was registered at clinicaltrials.gov (NCT05232084). The study included 43 female patients aged 18-65 years who were classified into the American Society of Anesthesiologist (ASA) I-II group and underwent elective, unilateral mastectomy and axillary lymph node dissection under general anaesthesia at Medipol Mega University Hospital between January 2022 and June 2023.

Patients with a thoracic region deformity or bleeding diathesis, those receiving anticoagulant treatment, those with infection in the block area, and those allergic to opioids and local anaesthetics were excluded from the study.

### Grouping and Randomization

Prior to their arrival in the operating room, the patients were randomly divided into 2 groups (Group 20 mL ESPB = Group 20; Group 30 mL ESPB = Group 30) using a computerized randomization program. Patients were informed about ESPB and the use of the patient-controlled analgesia (PCA) device, and they provided written informed consent.

### General Anaesthesia

Following patient transfer to the operating room, electrocardiography, peripheral oxygen saturation (SpO_2_), and non-invasive arterial blood pressure were continuously monitored. Intubation was then performed using an appropriately sized endotracheal tube. Anaesthesia was maintained by infusion of 0.01-0.1 µg kg^-1^ min^-1^ remifentanil and inhalation of 1-2% sevoflurane in a 50% oxygen/air mixture. The mechanical ventilator settings were adjusted to a tidal volume of 6-8 mL kg^-1^ and an end-tidal CO_2_ of 30-35 mmHg. All surgical procedures, including breast-conserving surgery and axillary lymph node dissection, were performed by the same team following the same protocol. For multimodal analgesia, all patients received 400 mg intravenous (IV) ibuprofen and 100 mg IV tramadol 30 minutes before the conclusion of the surgery. Additionally, ondansetron was intravenously administered to prevent postoperative nausea and vomiting. Participants who exhibited sufficient spontaneous breathing were extubated.

### ESPB Procedure

**Figure 1:**
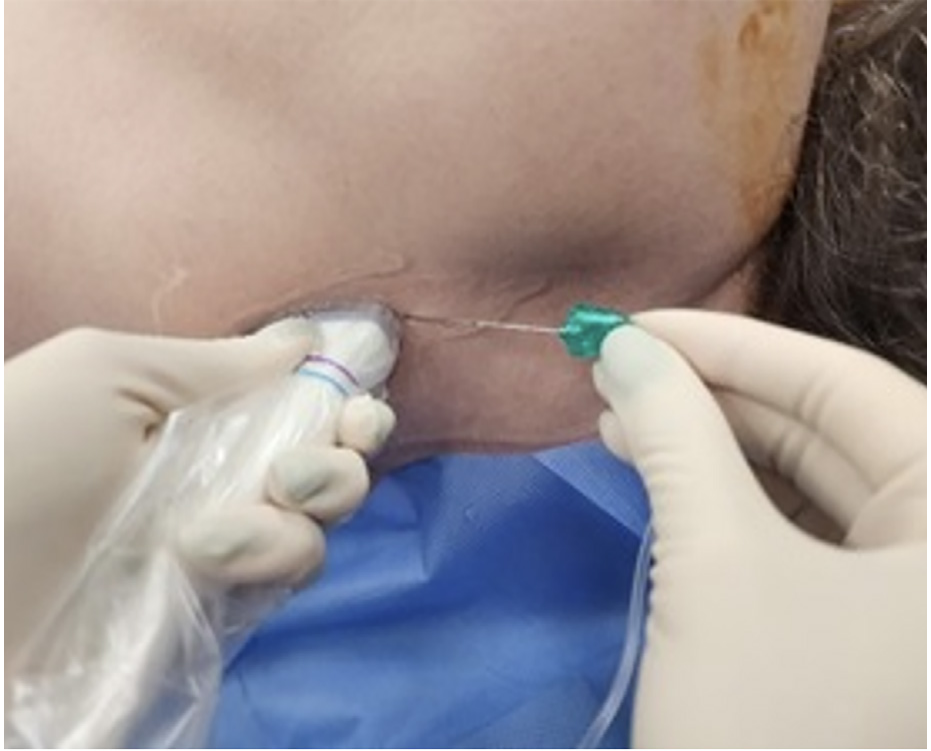
Patient, probe, and needle positions during block

Following the surgical procedure, ESPB was performed under general anaesthesia before extubation. During the ESPB, the patient was placed in the lateral decubitus position with the surgical side up during the ESPB (Figure 1).

**Figure 2:**
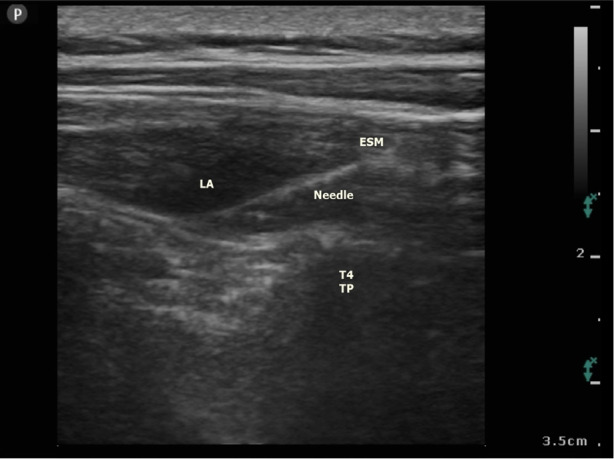
Sonographic images of the ESPB at the T4 vertebral level and local anaesthetic spread. ESM; erector spinae muscle; TP, transverse process, LA; local anaesthetic

A high-frequency linear transducer (11-12 MHz) and an 80-mm block needle (Braun 360°) were used. The transducer was located longitudinally over the TP of the T4 vertebrae. The muscles are seen on the hyperechoic TP, from top to bottom: the ESM, rhomboid major in the middle, and trapezius at the top. Using the in-plane technique, the block needle was moved forward in the craniocaudal direction, and five milliliters of regular isotonic injection were used to confirm the block position deep into the ESM. Following confirmation, Group 20 was given 20 mL of 0.25% bupivacaine, while Group 30 was given 30 mL of the same substance (Figure 2).

### Protocol for the Postoperative Management and Evaluation of Outcomes

Following surgery, patients were administered 400 mg of ibuprofen every 8 hours. Patients in all groups were connected to an IV PCA containing 10 µg mL^-1^ fentanyl, a 10 µg bolus without an infusion dose, and a 10-min lock-time protocol. An anaesthesiologist who was not involved in the study conducted a postoperative patient evaluation.NRS was used for postoperative pain evaluation (From 0/no discomfort to 10/the greatest amount of pain felt). Resting and dynamic NRS scores were evaluated and recorded at the 1^st^, 2^nd^, 4^th^, 8^th^, 16^th^, and 24^th^ hours. If the NRS score was ≥4, 0.5 mg kg^-1^ IV meperidine was administered as a rescue analgesic. The amount of rescue analgesic administered, the amount of PCA used, and any side effects, such as itchiness, nausea, and emesis, were recorded.

### Quantity of Samples and Statistical Evaluations

The G*Power tool (V.3.1.9) was used to determine the study sample size. Comparison of postoperative opioid usage was the main goal. A preliminary study involving eight patients included in each group (16 patients in total) revealed postoperative opioid consumption of 20 µg in Group 20 and 30 µg in Group 30. The standard deviations were determined as 13 µg and 11 µg, respectively. Based on 0.05 alpha (α) error and 0.20 β error, the minimum number of patients needed to be included in each group was 19 with 80% power.

The Shapiro-Wilk test was used to assess the normality of the data and distribution patterns of the variables. When test results showed that the data were normally distributed, the data were described using mean ± standard deviation and subjected to an Independent Samples t-test to evaluate group-wise differences in outcome parameters. Group differences were examined using the Mann-Whitney U test, and continuous data with a non-parametric distribution were characterized using the median and interquartile range. A value of p<0.05 was considered statistically significant. We used SPSS V.25 (SPSS, Chicago, IL, USA) for all statistical analyses.

## Results

**Figure 3:**
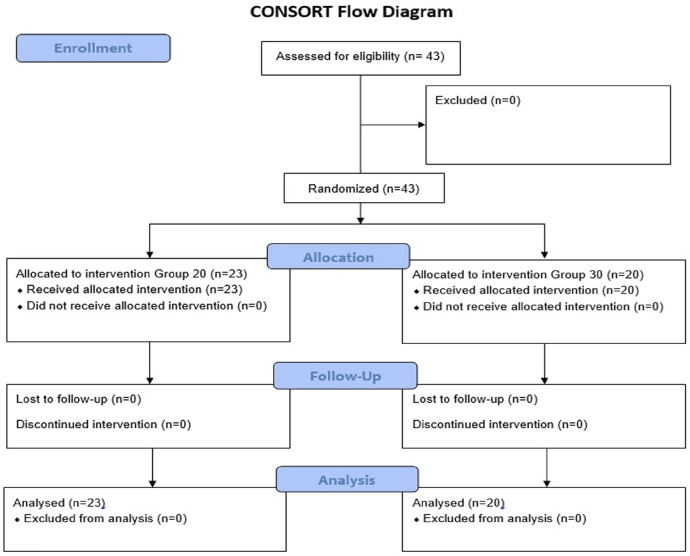
CONSORT flow diagram of the study

A CONSORT flow diagram was used for patient enrollment in the study (Figure 3). Each patient’s age, ASA score, height, weight, surgical duration, and anaesthesia duration are presented in Table 1. There were no discernible changes in demographic information, surgical duration, or anaesthetic duration in the groups.

Fentanyl use from the PCA device was evaluated at postoperative 0-8, 8-16, and 16-24 hours, and as total consumption in 24 hours. Postoperative fentanyl use showed no discernible variation between the groups. Nine patients in Group 20 and eight in group 30 required rescue analgesics. The extent of rescue analgesic use was similar between the groups (Table 2).

NRS scores were recorded in both groups at the 1^st^, 2^nd^, 4^th^, 8^th^, 16^th^, and 24^th^ hours. The static and dynamic NRS scores were similar between the groups (Table 3). No statistical difference was noted in terms of nausea, vomiting, or itching between the groups (Table 4).

## Discussion

This study compared the effectiveness of ESPB administered at different volumes (20 mL vs. 30 mL) for postoperative analgesia management after mastectomy and axillary lymph node dissection. Our results indicated no difference in postoperative analgesia management between 20 mL and 30 mL ESPB in participants undergoing breast surgery and axillary dissection. According to our results; there was no difference between groups in terms of fentanyl use from the PCA device, static/dynamic NRS, need for rescue analgesia, and adverse effects.After breast surgery, patients often experience significant pain due to extensive tissue manipulation and potential nerve damage. Effective pain management is crucial to facilitate recovery and improve quality of life,^1, 2^ particularly because postsurgical pain in these patients can lead to complications, such as delayed mobilization, prolonged hospital stays, and decreased patient comfort. Methods for analgesia include a combination of pharmacological and regional anaesthesia approaches. Pharmacologically, opioids, non-steroidal anti-inflammatory drugs, and acetaminophen are commonly used to manage acute postoperative pain, whereas regional anaesthesia techniques provide targeted pain relief and reduce the need for systemic medications. Although the most commonly used analgesic method for this purpose is opioids, these drugs have adverse effects. For this reason, regional anaesthesia methods have begun to take on prominence. Several regional anaesthesia techniques are used for the breast and axillary regions. A multimodal pain management strategy tailored to the individual patient’s needs is often the most effective approach for controlling pain following breast surgery.^1^

ESPB is an innovative plane-blocking technique used to manage pain, particularly in thoracic and abdominal surgeries, including breast surgery. This block involves the injection of a local anaesthetic into the plane at the depth of the ESM adjacent to the TP of the thoracic vertebrae.^3, 4, 5^ The mechanism of the ESPB hinges on the diffusion of the anaesthetic along the fascial planes and the subsequent spread of the drug to the dorsal and ventral rami of the spinal nerves. This diffusion effectively numbs the nerves responsible for transmitting pain signals from the surgical site. By blocking these nerves, the ESPB provides substantial pain relief and reduces the need for systemic analgesics, such as opioids, which can have significant side effects. This technique is valued for its simplicity, safety, and ability to cover a wide area of innervation, making it a versatile option for managing postoperative pain. In recent years, many studies on the ESPB mechanism have been published. In their article, Bailey and Uppal^11^ asked whether the ESPB is a silver bullet or overhyped. They stated that the possible mechanism of ESPB is an anaesthesia’s dissemination to the paravertebral and epidural spaces. Coppens et al.^12^ raised the question of whether ESPB is “Stranger Things” or a “paranormal block”. In their article, they explained the mechanism of action of ESPB as a “triple mechanism” involving dorsal, anterior, and paravertebral spread. The available literature indicates that all these mechanisms and diffusion/spread theories are volume-dependent. Tulgar et al.^13^ asked whether ESPB is “a miracle or self-persuasion,” and emphasized that the mechanism of action of ESPB actually changes when the needle touches different points of the TP and when the volume differs. Gadsden et al.^14^ compared the spread of different volumes of ESPB in a cadaver study. They reported that the spread increased with the applied volume, and they concluded that the ideal volume for ESPB was 30 mL. Ciftci et al.^15^ compared 20 mL and 30 mL ESPB in their cadaver study and stated that the spread was greater with 30 mL, but they reported no axillary spread with either volume. While cadaver studies have suggested that higher volumes may be more effective, our clinical study found that 30 mL and 20 mL volumes had comparable effects. We attribute this difference to factors such as tissue perfusion in living subjects versus cadavers and the influence of respiratory movement on local anaesthetic spread. Although cadaver studies are invaluable for regional anaesthesia techniques, the solution distribution in blood-perfused living tissue may follow distinct patterns. Additionally, the similar efficacy between volumes in our study may be related to the individual anatomical characteristics of the patients. Abdella  et al.^16^ evaluated the effect of different volumes (20 mL vs. 40 mL) of ESPB on the pain scores, craniocaudal spread, paravertebral spread, epidural spread, and clinical dermatomal spread in patients who underwent mastectomy. They reported no difference in pain scores, spread to the paravertebral area, epidural area, or exit point of the spinal nerve roots. However, in the high-volume applied group, the craniocaudal spread area was wider. In our study, there was no difference between the groups in terms of pain scores or opioid consumption. Zengin et al.^17^ conducted a clinical trial that compared 20 mL vs. 30 mL ESPB in patients who underwent thoracotomy. They concluded that 30 mL of ESPB provided more effective analgesia. Solmaz Demirel et al.^7^ compared 20 mL vs. 30 mL volumes of ESPB in patients who underwent breast surgery. They used 0.375% bupivacaine and reported no differences between the groups. Similarly, in our study, we found no difference between the effectiveness of 20 mL or 30 mL volumes of bupivacaine at a 0.25% concentration. On the other hand, the concentration is as important as the volume for the effectiveness of the block. Altıparmak et al.^18^ compared different concentrations of ESPB (0.375% vs 0.25% bupivacaine) and reported that higher is more effective than lower concentrations. In our study, different volumes with the same concentrations were compared.

### Study Limitations

Our study has some limitations. Our sample size was relatively small; therefore, different results may be obtained with studies involving a larger number of patients. Different results may also be obtained for volumes other than 20 mL and 30 mL. We were also unable to perform dermatome analysis; thus, further valuable results could be obtained by performing this analysis.

## Conclusion

According to our results, a similar analgesic effect was achieved by performing ESPB using 20 or 30 mL of local anaesthetic at the same concentration. NRS scores, opioid consumption, and incidence of adverse effects were similar between the groups.

## Ethics

**Ethics Committee Approval: **This prospective, randomized study was approved by the İstanbul Medipol University Non-Interventional Clinical Research Ethics Committee (approval no.: 997, dated: September 30, 2021).

**Informed Consent: **Patients were informed about ESPB and the use of the patient-controlled analgesia (PCA) device, and they provided written informed consent.

## Figures and Tables

**Table 1. Comparison of Demographic Data, Surgery and Anaesthesia Times Between Groups:** 

-	**Group 20 (n=23)**	**Group 30 (n=20)**	***P* value**
**Age**	51 (41-59)	60 (45-63)	0.247*
**ASA (I/II)**	11/12	7/13	0.395^†^
**Height (cm)**	165 (161-170)	157 (154-160)	0.565*
**Weight (kg)**	71 (65-80)	66 (58-79)	0.201*
**Duration of surgery (min)**	110 (93-129)	106 (97-127)	0.981*
**Duration of anaesthesia (min)**	85 (68-105)	80 (65-100)	0.687*

Values are expressed as mean ± standard deviation or numbers.

*P value was obtained by Mann-Whitney U test median (percentiles 25-75).

^†^*P *value was obtained by Pearson’s χ2 test (n).

ASA, American Society of Anesthesiologist.

**Table 2. Comparison of Postoperative Opioid Consumption and Rescue Analgesia Use Between Groups:** 

-	**Group 20 (n=23)**	**Group 30 (n=20)**	***P *value**
**PCA 0-8^th^ hour (µg)**	0 (0-10)	0 (0-20)	0.537
**PCA 8-16^th^ hour (µg)**	0 (0-0)	0 (0-10)	0.504
**PCA 16-24^th^ hour (µg)**	0 (0-0)	0 (0-0)	0.351
**PCA total (µg)**	0 (0-15)	0 (0-40)	0.647
**Rescue analgesia (number of patients)**	9	8	0.95^†^
**Dosage of rescue analgesia (mg)**	100 (100-100)	100 (62-100)	0.271

Data are expressed as median.

*P* value was obtained by Mann-Whitney U test median (percentiles 25-75).

^†^*P* value was obtained by Pearson’s χ^2 ^test (n).

PCA, patient-controlled analgesia.

**Table 3. Comparison of Static and Dynamic NRS Assessment Between Groups:** 

-	**Group 20 (n=23)**	**Group 30 (n=20)**	***P *value**
**Static NRS**			
1^st^ hour	0 (0-1)	0 (0-0)	0.221
2^nd^ hour	0 (0-2)	0 (0-3)	0.369
4^th^ hour	0 (0-1)	0 (0-2)	0.105
8^th^ hour	0 (0-1)	0 (0-0)	0.497
16^th^ hour	0 (0-0)	0 (0-0)	0.615
24^th^ hour	0 (0-0)	0 (0-0)	0.218
**Dynamic NRS**			
1^st^ hour	0 (0-2)	0 (0-0)	0.147
2^nd^ hour	0 (0-2)	1 (0-4)	0.529
4^th^ hour	0 (0-1)	0 (0-2)	0.142
8^th^ hour	0 (0-2)	0 (0-1)	0.295
16^th^ hour	0 (0-1)	0 (0-1)	0.975
24^th^ hour	0 (0-0)	0 (0-0)	0.120

Data are expressed as median (25-75^th ^percentile).

NRS, numerical rating score.

**Table 4. Comparison of Side Effects Between Groups:** 

-	**Group 20 (n=23)**	**Group 30 (n=20)**	**P value**
**Nausea**	6	6	0.775
**Vomiting**	2	2	1
**Itching**	4	6	0.473

*P* value was obtained by Pearson χ^2 ^(n) test.
